# Immununochemical Markers of the Amyloid Cascade in the Hippocampus in Motor Neuron Diseases

**DOI:** 10.3389/fneur.2016.00195

**Published:** 2016-11-08

**Authors:** Ulises Gómez-Pinedo, Rocio N. Villar-Quiles, Lucia Galán, Jordi A. Matías-Guiu, Maria S. Benito-Martin, Antonio Guerrero-Sola, Teresa Moreno-Ramos, Jorge Matías-Guiu

**Affiliations:** ^1^Neurobiology Laboratory, Faculty of Medicine, Neuroscience Institute, IdISSC, Hospital Clínico San Carlos, Universidad Complutense de Madrid, Madrid, Spain; ^2^Neurology Department, Faculty of Medicine, Neuroscience Institute, IdISSC, Hospital Clínico San Carlos, Universidad Complutense de Madrid, Madrid, Spain

**Keywords:** amyotrophic lateral sclerosis, frontotemporal dementia, amyloid precursor protein, Aβ peptide, AICD peptide, TDP-43, TAU protein, Fe65 protein

## Abstract

**Background:**

Several findings suggest that the amyloid precursor protein (APP) and the amyloid cascade may play a role in motor neuron disease (MND).

**Objective:**

Considering that dementia is one of the most frequent non-motor symptoms in amyotrophic lateral sclerosis (ALS) and that hippocampus is one of the brain areas with greater presence of amyloid-related changes in neurodegenerative diseases, our aim was to analyze the molecular markers of the amyloid cascade of APP in pathology studies of the hippocampus of autopsied patients with ALS and ALS–frontotemporal dementia (FTD).

**Methods:**

We included nine patients with MND and four controls. Immunohistochemical studies and confocal microscopy were used to analyze the expression of APP, TDP-43, pho-TDP-43, Aβ, APP intracellular cytoplasmatic domain (AICD) peptide, Fe65 protein, and pho-TAU in the hippocampus of seven patients with ALS, two patients with ALS–FTD, and four controls. These findings were correlated with clinical data.

**Results:**

Patients displayed increased expression of APP and Aβ peptide. The latter was correlated with cytoplasmic pho-TDP-43 expression. We also found decreased Fe65 expression. A parallel increase in AICD expression was not found. Patients showed increased expression of pho-TAU in the hippocampus. Findings were similar in patients with ALS and those with ALS–FTD, though more marked in the latter group.

**Conclusion:**

Post-mortem analyses showed that the amyloid cascade is activated in the hippocampus of patients with MND and correlated with cytoplasmic pho-TDP-43 expression. The number of intracellular or extracellular aggregates of Aβ peptides was not significant.

## Introduction

Amyotrophic lateral sclerosis (ALS) is a neurodegenerative disease affecting motor neurons in the brain, brainstem, and spinal cord. Its prevalence rate is estimated at 4–6 cases per 100,000 population (1). It has a variable clinical course, and onset is usually focal (more frequently spinal than bulbar). Life expectancy is estimated at 3–5 years on average. Although the cause is unknown, it has been linked to mutations in different genes even in the sporadic forms (2). Pathology studies in patients with ALS have shown that degeneration not only affects motor areas but also the dorsolateral prefrontal cortex, anterior cingulate, hippocampus, dentate gyrus (DG), parietal lobe, substantia nigra, cerebellum, amygdala, and basal ganglia (3).

The copresence of several neurodegenerative diseases in some patients has led to defining new groups of diseases, some of which are linked to certain genetic or molecular markers. For example, the association between ALS and certain forms of frontotemporal dementia (FTD) has been linked to TDP-43 positive cytoplasmic inclusions as well as to mutations in the gene encoding this protein or other related proteins, in both familial and sporadic forms whether these molecular changes are a cause or a consequence of neuronal degeneration is unclear (4–7). Although the association between ALS and Alzheimer disease (AD) has not been well studied, some authors have suggested that these entities may co-occur. Although the association between ALS and AD has not been well studied, some authors have suggested that these entities may co-occur (8–17).

Although patients with ALS may also present memory impairment and even dementia (18), only two pathology studies have analyzed the presence of changes compatible with AD. In a series of 30 patients with ALS, Hamilton and Bowser (19) found a prevalence of dementia of 24.1%. Around 30% of the patients displayed pathological changes associated with AD, especially amyloid and neuritic plaques in the hippocampus, which were more common in the DG and neocortex. These authors also found patients without dementia who showed AD lesions; Aβ deposition was present in up to 50% of cases. In that study, pathological changes of AD, especially neuritic plaques, were associated with older age and shorter survival time (20). In another study, Coan and Mitchell (20) analyzed 46 autopsies of patients with ALS and found that 22% of the cases met criteria for AD and 26% for FTD, 78% displayed neurofibrillary tangles, and 35% showed moderate increases in Aβ expression, especially in the amygdala, hippocampus, and the entorhinal and insular cortices. Likewise, ALS onset in patients who met the criteria for AD was more frequently bulbar; onset type had no impact on survival time. All patients with Aβ peptide accumulation showed greater prevalence of neurofibrillary tangles in the hippocampus and amygdala. Furthermore, the form of ALS reported in Guam residents is associated with abundant neurofibrillary tangles in addition to the classic neuropathological findings of the disease (21, 22).

Amyloid cascade is defined by the consequences of amyloid precursor protein (APP) cleavage after two successive proteolysis, which has served as the basis for the amyloid hypothesis of AD (23), and finally produces Aβ. While β- and γ-secretases promote Aβ formation, α-secretase has the opposite effect, which results in two pathways: the amyloidogenic and the non-amyloidogenic APP pathways, respectively. The last proteolysis in the amyloidogenic pathway produces two substrates: the peptides Aβ and the APP intracellular cytoplasmatic domain (AICD), which plays an important role in the transcriptional regulation of certain genes. The idea that certain pathological changes of AD may be present in patients with ALS or ALS–FTD suggests the possibility that they have common mechanisms (24), but it may also indicate that these changes are not primary (25). Patients with ALS do not generally present clinical changes as intense amnestic symptoms suggesting that the hippocampus could be significantly affected, contrary to what occurs in AD. Taking into consideration that the hippocampus is one of the areas of the brain most affected by molecular changes associated with AD, we analyzed the expression of proteins linked to the amyloid cascade in the hippocampus of autopsied patients with ALS or ALS–FTD.

## Materials and Methods

### Selection and Study of Patients and Controls

We analyzed the autopsies of nine patients that were included in the ALS registry of our neurology department. This registry follows a protocol for diagnosing and treating ALS that includes clinical assessment, electromyographic and neuroimaging studies, and blood and CSF analyses, as well as criteria for assessing FVC and using BiPAP, and indicating gastrostomy and riluzole (26). These patients, who died between 2006 and 2012, met the El Escorial revised and Ludolph criteria for ALS (27, 28). The following clinical variables were included in our analysis: age, sex, disease duration, clinical form, whether riluzole was administered, and clinical data related to potential cognitive impairment. Data were gathered from the ALS registry by thoroughly reviewing clinical histories and conducting telephone interviews with the patients’ relatives. Eight of the patients included in our study had died of respiratory failure at the terminal stage of ALS. The remaining patient died due to cardiac arrest upon admission after attending the hospital due to progressive dysphagia and moderate bulbar involvement and being diagnosed with ALS. Our controls were four patients who died at a hospital due to a non-neurological disease. They were included because their families had consented to donation of the body. None of them had a history of neurodegenerative diseases. We reviewed their medical records to rule out a history of neurological disease. One of our controls was donated by another center; we were therefore unable to review the medical history but confirmed that the patient had not died from a neurological cause and had no history of neurodegenerative disease.

### Autopsy Procedure and Preparation and Storing of Biological Material

Autopsies were performed within 2–6 h after death following our hospital’s standard protocol and in compliance with Spanish regulations for this procedure. We followed the standard method: opening the cranial cavity and severing the upper end of the spinal cord at the foramen magnum. To separate the hemispheres, we cut right along the midline of the corpus callosum and prepared them for later sectioning. Tissue samples were fixed in 10% buffered formalin [phosphate-buffered saline (PBS); 0.1M, pH 7.35]. The hemisphere allocated to histological and immunohistochemical analyses was sectioned into coronal slices (maximum slice thickness = 1 cm), which were placed on a flat surface in order from the frontal pole to the occipital pole. The nervous system analysis included weighing the brain, examining its macroscopic morphology, and conducting a microscopic study, using conventional techniques. In all cases, the study included hematoxylin-eosin and Nissl staining. Additional techniques were used at the discretion of the pathologist, including Congo red and silver staining to determine the presence of such macroscopic and microscopic alterations as atrophy, senile plaques, eosinophilia, or neuronal loss. Thioflavin-S staining was used for all cases and controls.

### Study of the Hippocampus

Once the tissue for microscopic analysis had been adequately prepared, paraffin blocks were sectioned to obtain the hippocampus, which should include at least the following areas: CA1, CA2, CA3, and DG. All tissue samples were embedded in paraffin following the protocol established by the pathology department at our hospital and subsequently sectioned to study the cytoarchitecture of the hippocampus. We used Braak staging, which was developed by Braak and Braak (29), to assess pathological findings.

### Immunohistochemical Study

Tissue was sectioned into 6-μm slices using a microtome (Leica). Slices were deparaffinized and thoroughly washed with PBS 0.1M. Epitopes were unmasked in a 10-mM sodium citrate buffer with a pH of 6 at 96°C for 30 min. Samples destined for APP immunostaining were additionally incubated for 20 min in formic acid v/v. Following this, all samples were incubated in a blocking solution (PBS, 0.2 Triton X-100, 10% normal goat serum) for 1 h. After that, tissues were incubated in primary antibodies diluted in PBS for 24 h. After incubation with primary antibodies, sections were washed with PBS and incubated in the appropriate HRP or Alexa-Fluor secondary antibody (see Table S1 in Supplementary Material). For APP and phospho TAU antibodies, goat anti-rabbit HRP was used, the sections were stained brown with a DAB-peroxidase solution of 0.03% diaminobenzidine and 0.01% H_2_O_2_, and counterstained with Nissl staining. They were subsequently mounted in DPX and observed under a ZEISS microscope. For the immunofluorescence study of the other markers, tissue sections were thoroughly washed and incubated with the appropriate Alexa-Fluor antibody for 24 h after incubation with primary antibodies. After thoroughly washing the sections, they were mounted in ProLong Gold reagent with DAPI (Molecular Probes, Invitrogen) and observed in an Olympus confocal microscope AF2000. Five hippocampal slides were used, those containing CA1–3 and DG (separated by at least 200 μm each, to avoid double quantifications), and the quantitative study included the analysis of 10 different fields for each of the analyzed antibodies; the result was the mean of the 10 measurements. In the cases where the unit of measurement was the amount of labeling per field [optical density (OD)], we used Image J software version 1.46r, developed by the National Institutes of Health. In the cases where inclusions were assessed, these were quantified based on the number of stained inclusions that were found in neurons divided by the mean number of neurons per field (percentage of cells in 500 μ^2^).

### Statistical Analysis

Statistical analysis was performed using SPSS statistical software version 20.0. Results were represented graphically using GraphPad Prism version 5.0. Data are expressed as mean ± SD. Means were compared using the non-parametric Mann–Whitney *U* test due to the small sample size. Graphs were created using the program mentioned above. Statistical significance was set at *p* < 0.05.

## Results

### Description of the Study Sample

Our study included nine patients (seven with ALS and two with ALS–FTD) and four controls. Five patients were men (56%), and four were women (44%). Mean age at diagnosis was 64.56 ± 16.8 years, and mean age at death was 65.33 ± 15.7 years. Regarding the controls, there were three men (75%) and one woman (25%), with a mean age at death of 68.75 ± 14.86 years. Six of the nine patients had an additional disease: one had a history of stroke, one had ulcerative colitis, two had a history of depression, one presented essential tremor, and the remaining one had REM sleep behavior disorder. Only one of the patients had a first-degree relative with a history of dementia of unknown origin. Likewise, only one patient presented a familial form of ALS. This patient, who had a non-pathological SOD1 mutation, had been included in a previous study (30). Mean time elapsed from onset of motor/bulbar symptoms to diagnosis of ALS was 6.44 ± 3.9 months (not including the two cases with ALS–FTD). Disease onset was bulbar in five patients (56%) and spinal in the remaining four (44%). Seven patients (78%) had received riluzole and six (67%) required permanent mechanical ventilation; mean time elapsed from diagnosis to indication of mechanical ventilation was 9 months. Five patients (56%) were being fed by gastrostomy. Mean time elapsed from onset of motor/bulbar symptoms to death was 17.6 ± 16.5 months (we did not count the time the two patients with FTD had presented impairment linked to FTD before ALS onset) and 11.11 ± 15.6 months from diagnosis to death. Cognitive disorders were only reported in the patients with ALS–FTD. Patient histories and clinical characteristics are shown in Table S2 in Supplementary Material.

### Description of General Pathological Findings in the Autopsies

Table S3 in Supplementary Material summarizes the results of the neuropathology study and shows the fixed brain weight for each subject. No significant differences were found in brain weight between patients with ALS (1220 ± 147 g) and controls (1290 ± 72.73 g; *p* = 0.244). Neuropathological findings resulted in a diagnosis of motor neuron disease (MND) in all patients. From a macroscopic point of view, precentral gyrus atrophy was seen in patients eight and nine and none of the controls. Likewise, two patients, and none of the controls, displayed thinning of the ventral roots of the spinal cord; all patients showed considerable neuronal loss in the anterior horn of the spinal cord. One of the patients exhibited loss of motor neurons in the medulla oblongata. One patient displayed numerous neurofibrillary tangles and amyloid plaques, while another showed moderate extracellular amyloid deposition. Ubiquitinated inclusions were found in the hippocampus and spinal cord of five patients. The mean number of neurons with ubiquitin-positive inclusions was 0.5 ± 0.58 in controls and 7.43 ± 2.7 in patients (*p* = 0.007), or 7.67 ± 0.8 if we exclude the patients with FTD (*p* = 0.01) (data expressed in 500 μm^2^ field).

### Cytoplasmic Phosphorylated TDP-43 Expression Is Increased in the Hippocampus of Patients with ALS and ALS–FTD

TDP-43 is a transcription factor that is located in the nucleus of cells. In MNDs, this protein is cleaved and translocated to the cytoplasm, where it may be phosphorylated. TDP-43, like ubiquitin, can be observed in cytoplasmic inclusions, leading to the idea of “TDP-43 pathology,” which encompasses such diseases as ALS and ALS–FTD. This protein is also found in the cytoplasm of neurons in patients with AD. In our sample, the mean percentage of expression of cytoplasmic phosphorylated TDP-43 (pho-TDP-43) in the hippocampus was higher in patients than in controls, which confirms that this area is affected. The area of the hippocampus showing the greatest TDP-43 expression was CA1, followed in decreasing order by GD, CA3, and CA2 (data not shown). The mean percentage of hippocampal cells with TDP-43-positive inclusions was 20.15 ± 10.46 (range 6.5–33.34) in the patient group and 1.62 ± 1.45 (range 0–2.67) in the control group. All patients displayed increased pho-TDP-43 expression, compared to controls; differences between patients and controls were statistically significant (*p* = 0.0028). Comparing ALS patients with ALS–FTD patients is not feasible since our sample included only two patients with ALS–FTD; however, higher pho-TDP-43 expression was observed in the ALS–FTD group. These data can be seen in Figure S1 in Supplementary Material.

### APP Expression Is Increased in the Hippocampus of ALS and ALS–FTD Patients but Is Not Correlated with TDP-43 Expression

**Figure 1:**
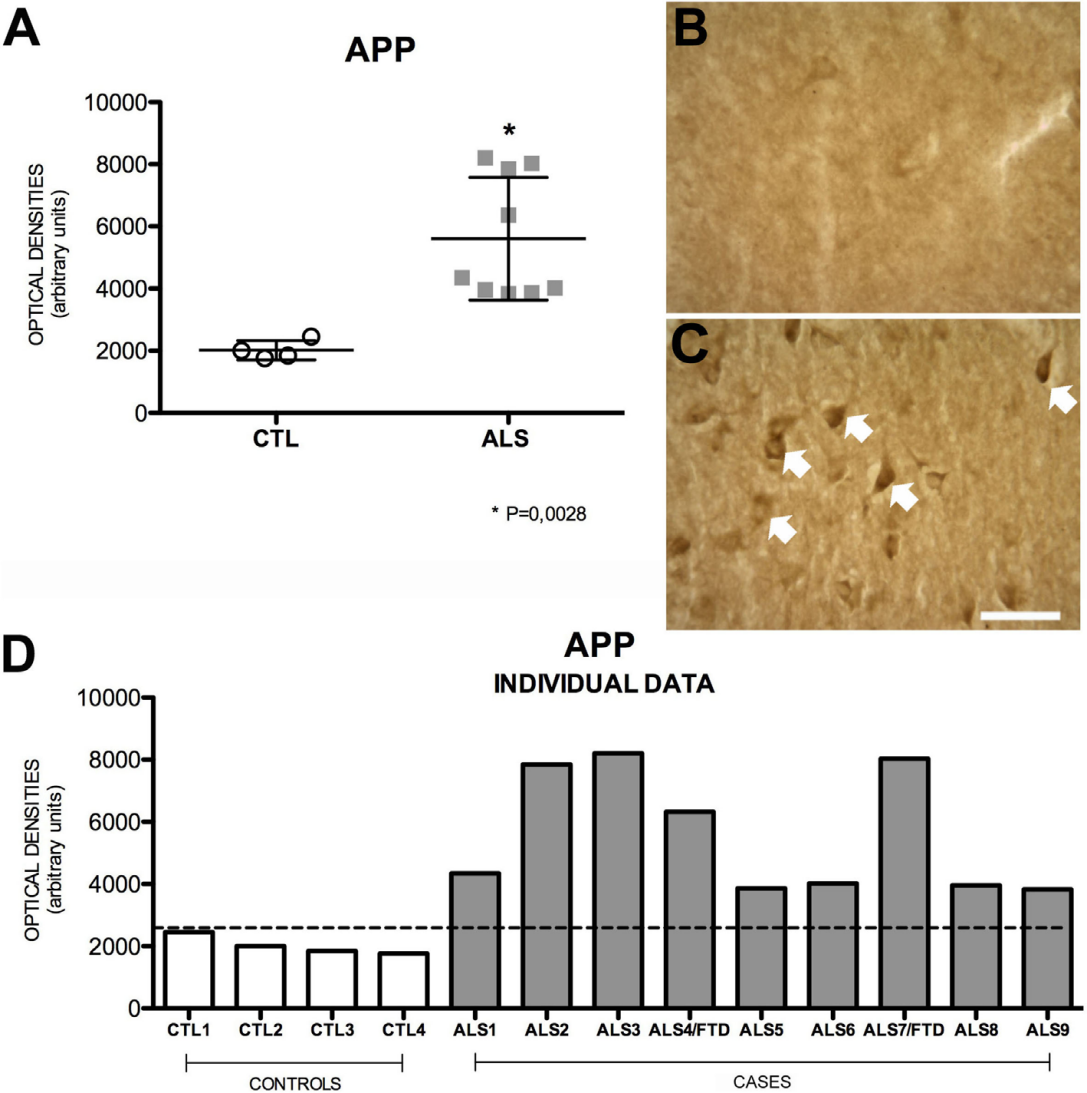
**APP expression (A) in mean values ± SD and individual values for controls and patients [(A,D), respectively] and immunohistochemistry images from controls (B) and patients (C)**. Patients show a statistically significant increase (*) in APP expression compared to controls, which may indicate that the amyloid cascade of APP is activated as a protective mechanism against the pathological effects of ALS. Photomicrographs **(B,C)** show the differences in labeling between controls and patients; as can be observed, APP expression (arrows) is greater in patients **(C)**. **(D)** Shows the individual data corresponding to patients and controls; APP expression is greater in patients than in controls, regardless of whether they had ALS or ALS–FTD. Scale bar: 50 μm. The dotted line indicates the significance threshold. The graphs display mean optical density (arbitrary units).

**Figure 2:**
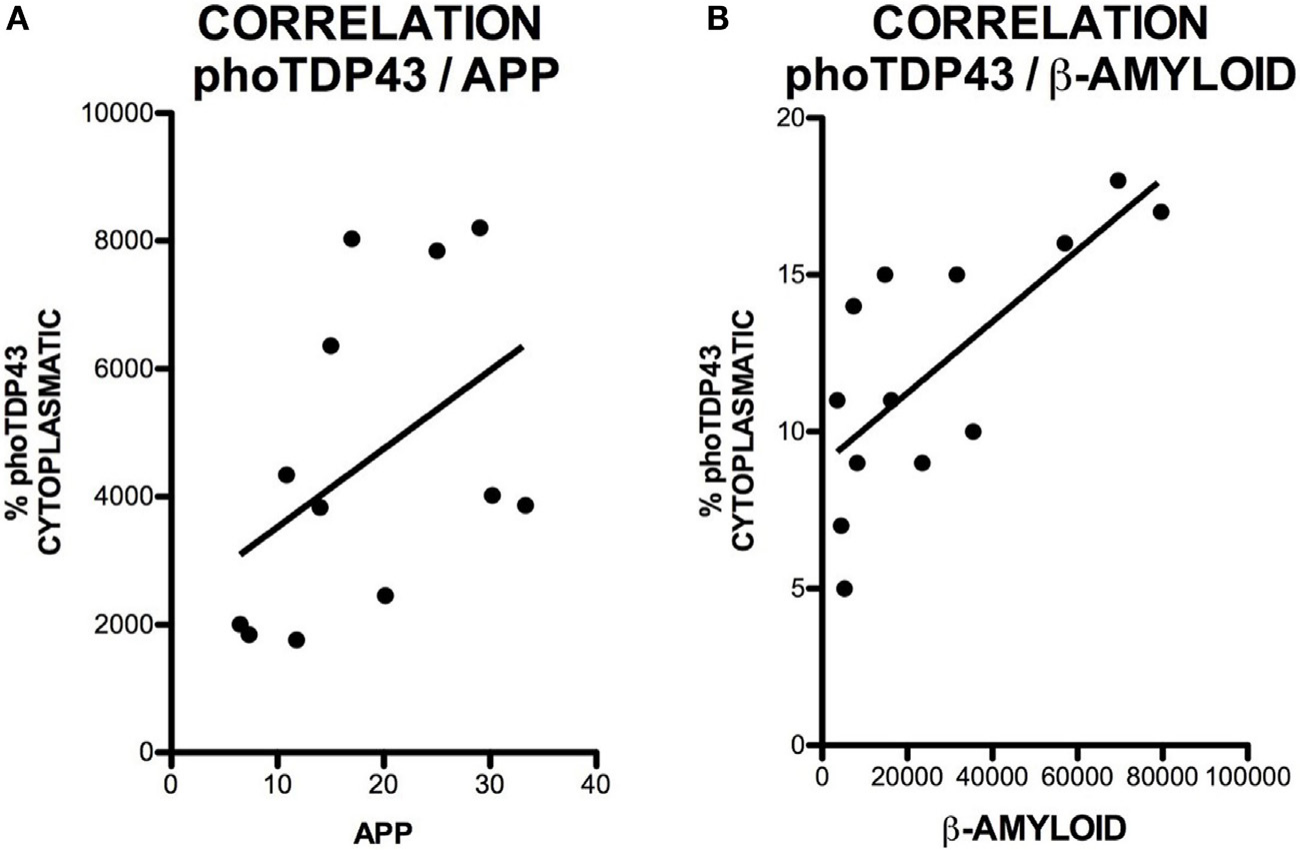
**Correlation between cytoplasmic pho-TDP-43 expression and APP expression (A) and between cytoplasmic pho-TDP-43 expression and Aβ expression (B)**. No correlation was found between pho-TDP-43 and APP. We observed a moderate correlation between pho-TDP-43 and Aβ.

Amyloid precursor protein is a ubiquitous transmembrane, type-1, integral glycoprotein of 110–130 kDa that is extensively expressed in human tissues. In the CNS, some functions attributed to APP are neurite outgrowth and synaptogenesis, protein trafficking along axons, cell adhesion, calcium metabolism, and signal transduction (31). Mean APP expression was higher in the hippocampus of our patients [4345 ± 1975 (range 3832–8204) OD] compared to controls [1925 ± 309 (range 1761–2458) OD]. APP expression was increased in all patients; differences between patients and controls were statistically significant (*p* = 0.0028). APP expression was greater in patients with ALS–FTD. These data are summarized in Figure 1. APP expression in the hippocampus was not correlated with phospo TDP-43 expression (*r* = 0.20), as shown in Figure 2A, and APP did not co-localize with TDP-43 in the cytoplasm (data not shown). Considering that APP plays an important role in cell survival, increased APP expression may be a cell response to neurodegeneration. However, the hippocampus is not clinically affected in ALS; the correlation found between APP and TDP-43 supports this hypothesis. The amyloid cascade of APP is active in the hippocampus in patients with MND and correlates with pho-TDP-43 expression.

**Figure 3:**
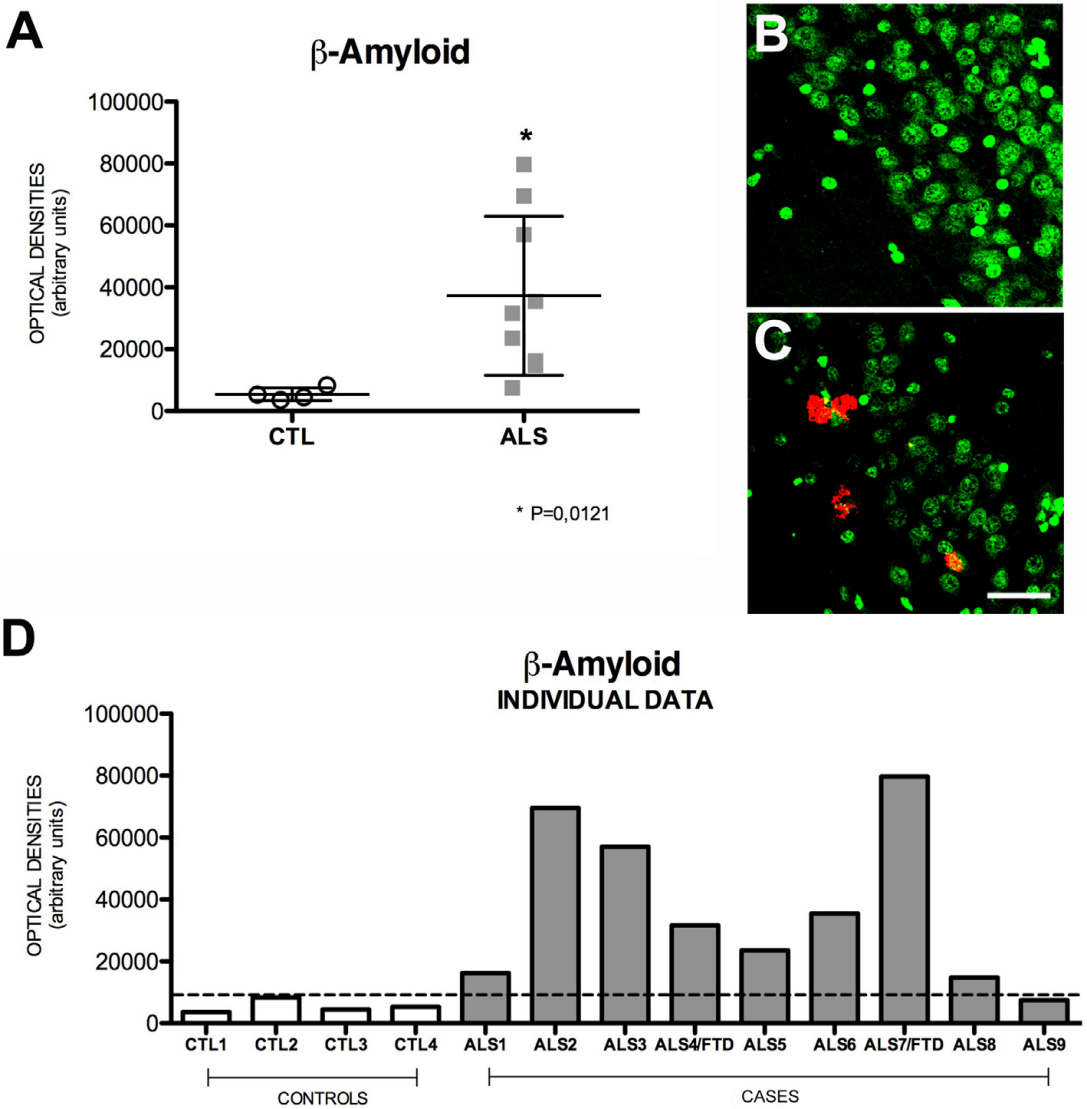
**Aβ expression (A) in mean values ± SD and individual values for controls and patients [(A,D), respectively] and immunohistochemistry images from controls (B) and patients (C)**. Patients displayed a statistically significant (*) increase compared to controls, which suggests that Aβ levels are higher in the hippocampus of patients with ALS. Photomicrographs **(B,C)** (dentate gyrus) show the differences in labeling between controls and patients; as can be observed, expression of Aβ-positive plaques is greater in patients **(C)** than in controls **(B)**. **(D)** Shows the individual data corresponding to patients and controls. Aβ expression is greater in patients; the highest value corresponds to a patient with ALS–FTD. Scale bar: 50 μm. The dotted line indicates the significance threshold. The graphs display mean optical density (arbitrary units).

The successive activity of two proteolytic processes, involving β- and γ-secretase, respectively, produces Aβ peptides, mainly Aβ40 and Aβ42. These may form oligomers and insoluble fibrils that accumulate both intracellularly and in extracellular amyloid plaques. This latter represents the pathological substrate for AD since Aβ peptide secretion plays a role in regulating neurotransmitter release in the synapses (32). Increased intracellular Aβ expression indicates greater amyloid cascade activity. Mean intraneuronal Aβ expression in the hippocampus was higher in patients than in controls: 32,261 ± 25,720 (range 7431–79,676) OD vs. 5393 ± 2048 (range 3542–8270) OD. All but one patient showed increased Aβ expression; differences between patients and controls were statistically significant (*p* = 0.0056). Greater APP expression was observed in one of the patients with ALS–FTD. These data are shown in Figure 3. The areas of the hippocampus showing increased intraneuronal Aβ expression were, in descending order, CA1, GD, CA3, and CA2 (data not shown). Although scarce, we observed Aβ deposits in the form of dense, diffuse plaques. Similar to what occurred with APP and cytoplasmic pho-TDP-43, Aβ expression and cytoplasmic pho-TDP-43 expression were moderately correlated (*r* = 0.530) (Figure 2B) but did not co-localize (data not shown). This correlation suggests that there is a link between cytoplasmatic pho-TDP-43 and activation of the amyloid cascade of APP in the hippocampus of patients with MND.

### Expression of AICD Is Variable in MND

**Figure 4:**
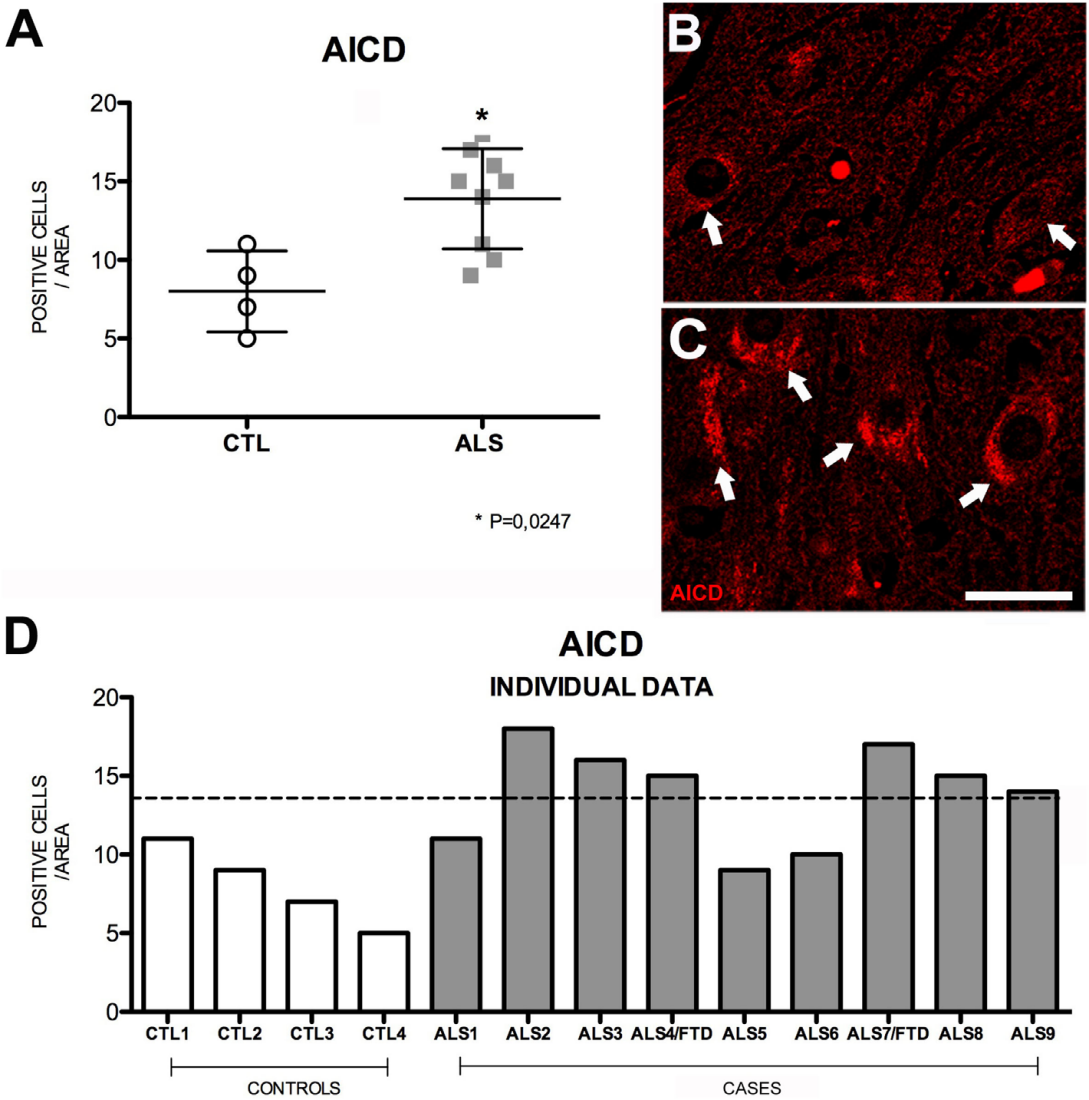
**AICD expression (A) in mean values ± SD and individual values for controls and patients [(A,D), respectively] and immunohistochemistry images from controls (B) and patients (C)**. Patients displayed a statistically significant (*) increase compared to controls, which indicates that AICD levels are higher in our patient sample. Photomicrographs **(B,C)** show the differences in labeling between controls and patients; AICD expression (arrows) is greater in patients **(C)** and low in controls (arrow). **(D)** Shows individual data for patients and controls. AICD expression is increased in patients: six patients (both with ALS and ALS–FTD) show higher values than those of controls. Scale bar: 20 μm. The dotted line indicates the significance threshold. The graphs present the percentage of cells with immunopositive inclusions in 500 μ^2^.

Amyloid precursor protein intracellular cytoplasmatic domain, the substrate common to both APP pathways, results from the activity of γ-secretase on sAPPα or sAPPβ; these soluble intermediate fragments are generated by α- and β-secretase, respectively (33, 34). This peptide forms a complex with adaptor protein Fe65 and histone acetyltransferase Tip60, and this complex plays an important role in regulating transcription of the genes encoding such proteins as APP, β-secretase, KAI1 (CD82), neprilysin, and p53 (35). In our sample, mean AICD expression in the hippocampus of patients was significantly raised compared to controls (*p* = 0.02); however, the expression was not elevated in three patients (Figure 4). The mean number of hippocampal cells showing AICD expression per area analyzed was 15 ± 3.18 (range 9–18) in patients and 8 ± 2.58 (range 5–11) in controls; patients with ALS–FTD displayed even higher numbers. These data are shown in Figure S2 in Supplementary Material. Wang et al. (36) demonstrated that AICD binds to and co-localizes with TDP-43 in the nucleus of cultured HEK293 cells. In our study, we neither found a significant correlation between AICD expression and cytoplasmic pho-TDP-43 expression nor did we observe co-localization between the two (data not shown).

### Fe65 Expression Is Lower in Patients with MND

**Figure 5:**
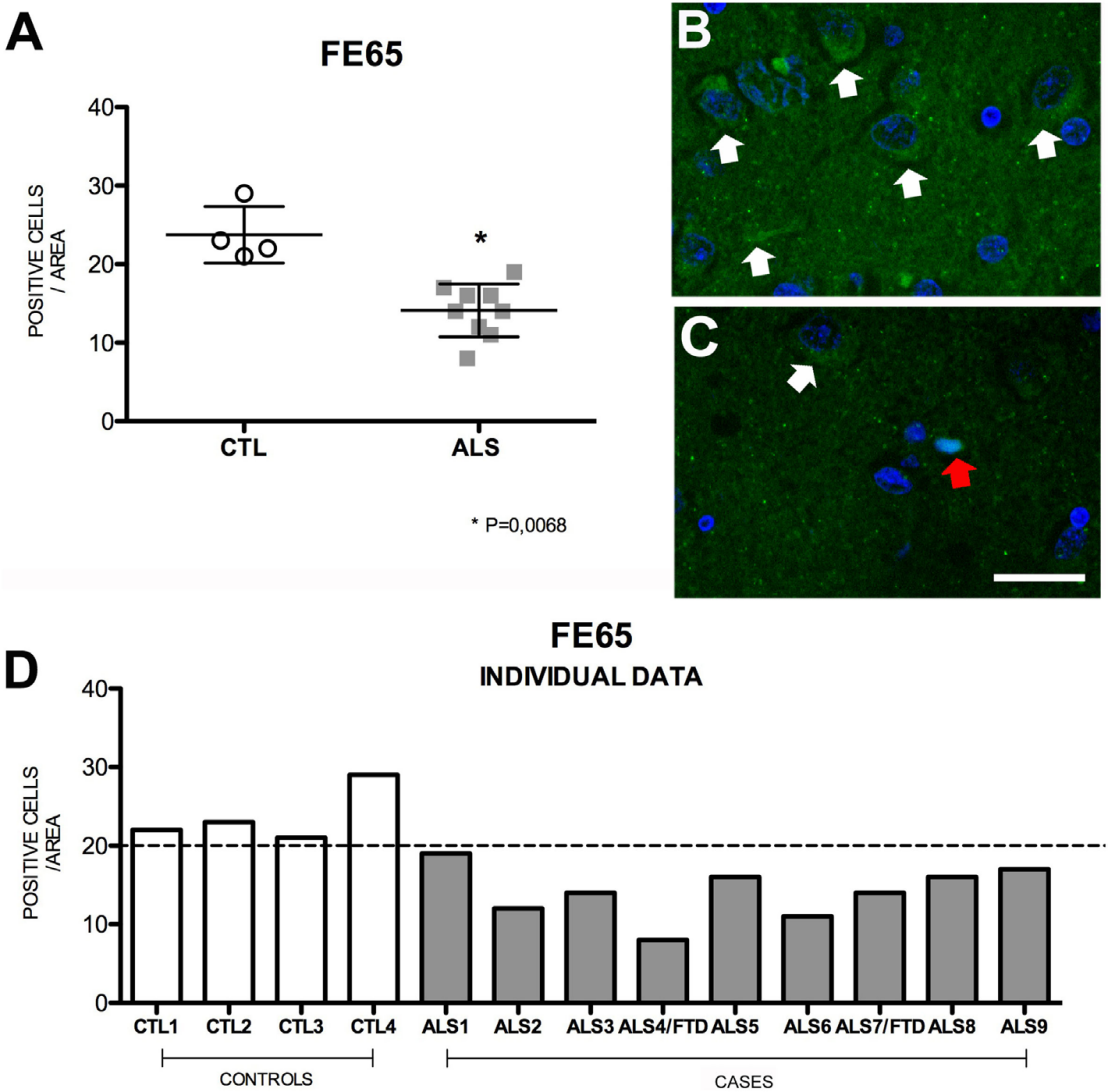
**Fe65 expression (A) in mean values ± SD and individual values for controls and patients [(A,D), respectively] and immunohistochemistry images from controls (B) and patients (C)**. Patients displayed a statistically significant (*) decrease in Fe65 expression compared to controls, which indicates that Fe65 levels are lower in our patient sample. Photomicrographs **(B,C)** show the differences in labeling between controls and patients; Fe65 expression is greater in patients **(C)** than in controls **(B)**, who display mild, diffuse labeling (arrow) and show nuclear expression (red arrow). **(D)** Shows individual data for patients and controls: controls display increased Fe65, and all patients show values below the statistical mean. Scale bar: 50 μm. The dotted line indicates the significance threshold. The graphs present the percentage of cells with immunopositive inclusions in 500 μ^2^.

Highly expressed in the hippocampus (37), Fe65 is an adaptor protein that is thought to play a crucial role in modulating the amyloid cascade of APP (38–42). Fe65 promotes the APP cascade by increasing Aβ production; this mechanism is attenuated by Fe65 phosphorylation (43). Mean Fe65 expression in hippocampal cells was significantly lower in patients [14 ± 3.37 (range 8–19)] than in controls [22.5 ± 3.37 (range 21–29)] (*p* = 0.018). None of the patients presented levels similar to those found in controls. Within the patient group, Fe65 expression was higher in patients with ALS–FTD. These findings (Figure 5) suggest that decreased Fe65 expression is probably due to phosphorylation in an attempt to decrease Aβ production. Cell labeling of Fe65 was at times weak or diffuses in the nucleus. The areas with greater positive immunoreactivity were, in descending order, DG, CA1, CA3, and CA2. Fe65 expression is inversely correlated with that of APP, AICD, and TDP-43 (Figure S3 in Supplementary Material), which supports the hypothesis that the amyloid cascade is activated in the hippocampus of patients with MND.

### Pho-TAU Is Expressed to a More Marked Degree in the Hippocampus of Patients with ALS

**Figure 6:**
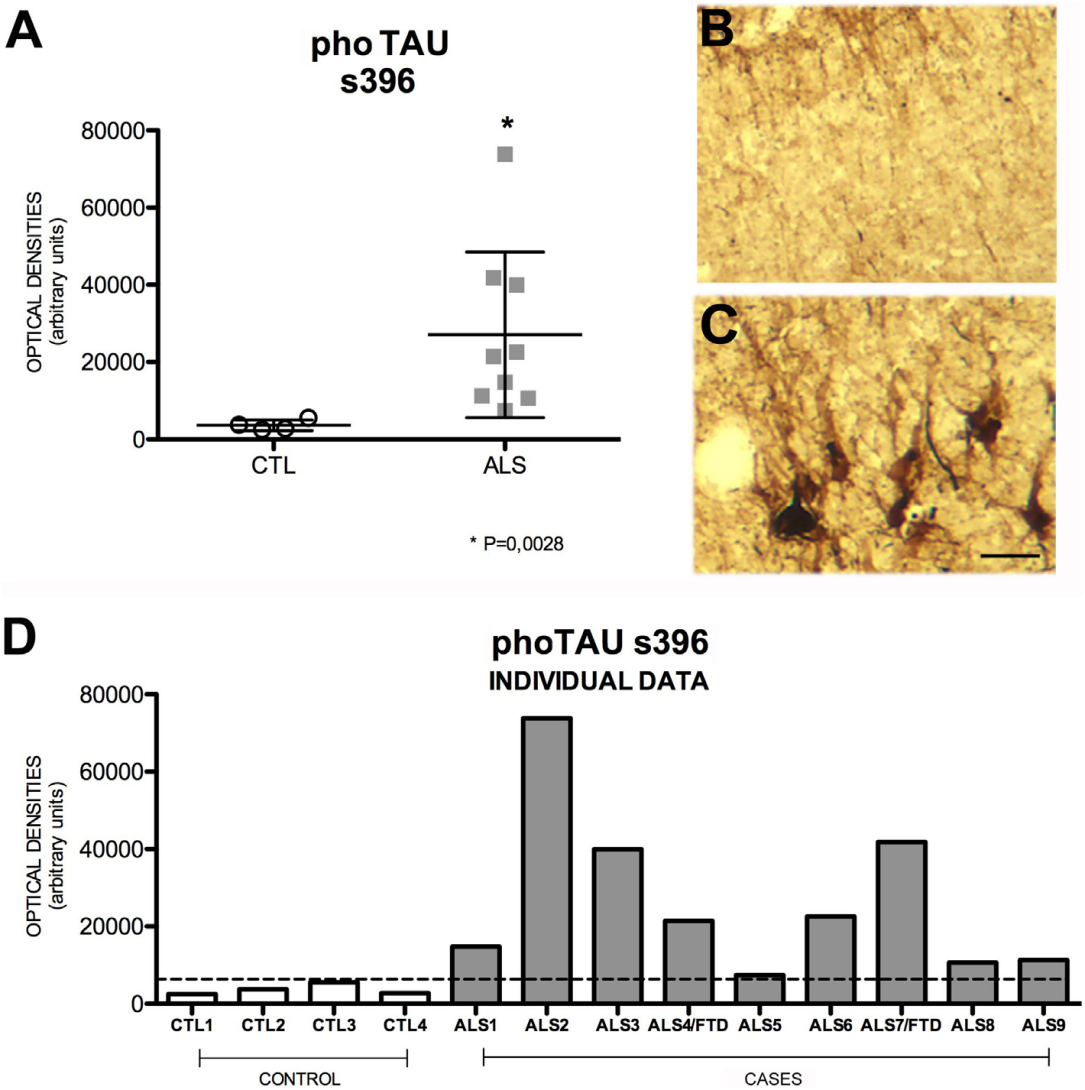
**Pho-TAU s396 expression (A) in mean values ± SD and individual values for controls and patients [(A,D), respectively] and immunohistochemistry images from controls (B) and patients (C)**. Patients display a statistically significant (*) increase in pho-TAU s396, which may indicate that the mechanisms of degeneration and microtubule-mediated axonal transport are affected. This means that levels of pho-TAU s396 are raised in ALS. Photomicrographs **(B,C)** (hippocampal area CA1) show the differences in labeling between controls and patients; as can be observed, expression of pho-TAU is much higher in the neurons of patients **(C)** than in those of controls **(B)**. **(D)** Shows the individual data corresponding to patients and controls. Pho-TAU s396 expression is greater in patients. Scale bar: 50 μm. The dotted line indicates the significance threshold. The graphs display mean optical density (arbitrary units).

TAU is overexpressed in patients with AD and other neurodegenerative diseases. TAU phosphorylation and aggregation is the molecular basis of neuritic plaques (44, 45). In our study, mean cell expression of hippocampal pho-TAU s396 was significantly increased in patients compared to controls (*p* = 0.002); none of the patients showed similar levels to those displayed by controls. Patients had a mean OD of 21,431 ± 21,455 (range 7431–73,844) and controls, 3278 ± 1378 (2481–5534). Pho-TAU s396 expression, which was slightly greater in patients with ALS–FTD (Figure 6), was shown to be inversely correlated with AICD expression (*r* = 0.570) (Figure S4 in Supplementary Material). A possible explanation for this correlation is provided by a study in which AICD-overexpressing transgenic mice showing no increase in Aβ levels displayed greater TAU expression and protein aggregation (46). The explanation for this is that AICD peptide upregulates GSK-3β expression, GSK-3β activation, and consequently TAU phosphorylation in rat neuronal cultures (47). This suggests that AICD induces TAU expression.

### Correlations with Clinical Profile and Progression

Despite the small number of patients included in our study, we correlated immunopathological findings with clinical characteristics and progression. These data are shown in Figure S5 in Supplementary Material. There is a statistically significant inverse correlation between total TDP-43 levels and time elapsed from diagnosis to death (*r* = 0.75), and between the percentage of cytoplasmic TDP-43 and time to indication of mechanical ventilation (*r* = 0.69). According to this latter correlation, greater levels of cytoplasmic TDP-43 are associated with a poorer prognosis. Likewise, increased expression of pho-TDP-43 and AICD are associated with bulbar onset (pho-TDP-43: 16.20 vs. 11.35% of cells with TDP-43-positive inclusions; AICD: 15.8 vs. 11.5, mean number of cells per analyzed area). Three patients showed increased pho-TAU s396 expression, which was associated with increases in AICD. Pho-TAU s396 overexpression was associated with more advanced Braak stages and greater quantities of thioflavin-S stained fibers.

Comparing immunopathology findings with the clinical forms of the disease showed that bulbar forms were associated with greater AICD (*p* = 0.031) and TDP-43 (*p* = 0.020) expression. We observed no significant differences in the expression of the remaining markers. Despite the small number of patients with ALS–FTD, the percentage of cytoplasmic TDP-43 was found to be greater in that subgroup (*p* = 0.0475).

The expression profile of markers showed no significant differences between patients with survival times shorter than 12 months and those with longer survival times. Higher levels of TDP-43 were correlated with shorter survival times (*r* = 0.753), as shown in Figure S5 in Supplementary Material.

## Discussion

There are many studies in the literature suggesting that neurodegenerative diseases share molecular characteristics; however, few studies have addressed this hypothesis with regard to AD and ALS (8, 19, 20). Findings from different studies point to certain common mechanisms. For example, Aβ accumulation has been found in the spinal cords of patients with both the familial and sporadic forms of ALS (48) as well as in the skin and muscles of ALS patients (49, 50), and biomarkers linked to the amyloid cascade have been found in the CSF of patients with ALS and FTD (49). In addition, increased APP expression has been observed in spinal cord motor neurons in experimental models in the early stages of ALS (51–54), and some experimental models of AD have displayed extracellular Aβ plaques in motor neurons, similar to those found in humans with AD (55). Likewise, increased Aβ expression has been found in affected motor neurons and the surrounding glial cells in SOD1^G93A^ mouse models; genetic ablation of APP in these mice reduces motor neuron degeneration (56). These findings are the reason for our interest in understanding biomarker expression in the amyloid cascade of APP and the connection with TDP-43 in patients with ALS.

Some clinical data, especially cognitive and neuroimaging findings, suggest that the hippocampus is affected in ALS (57, 58). Some authors have even proposed that hippocampal involvement in ALS has a different, less marked, pattern to that associated with AD degeneration (59, 60). Our aim was to study molecular alterations in the hippocampus, since this area shows no clinical changes in ALS but is greatly affected in AD patients. We found increased expression of cytoplasmic TDP-43 and pho-TDP-43, which confirms that ALS affects the hippocampus at a molecular level. Increased levels of TDP-43 in the hippocampus have been reported in previous studies, although they were linked to long disease progression times (61, 62). This is not applicable to our patients, who presented a short mean survival time (17.6 months from symptom onset). In any case, the presence of both cytoplasmic TDP-43 and pho-TDP-43 indicates that the mechanisms of neurodegeneration are active in the hippocampus of patients with MND.

Our study shows that the amyloid cascade of APP is activated and expressed in the hippocampus *via* its molecular markers in both ALS and ALS–FTD, as we found increased expression of APP and Aβ peptides, and even pho-TAU s396 overexpression. This increase in Aβ peptides is correlated with the expression of cytoplasmic pho-TDP-43 peptides. These data may suggest that APP expression and amyloid cascade activation are a response to molecular changes caused by MND: increased APP expression may be a mechanism of cell survival.

Our patients showed reduced Fe65 expression, probably due to the fact that Fe65 binds to AICD to downregulate APP expression. The hypothesis that Fe65 forms a complex with AICD is reinforced by the fact that, in some patients, increases in Fe65 expression were not present as would be expected considering that Fe65 production is simultaneous to that of Aβ after γ-secretase activity. We also found increased expression of pho-TAU in the hippocampus in all cases. Although it has been suggested that increased TAU expression is linked to AICD production (63), we found no correlation between the two. It is therefore most likely due to increased intraneuronal Aβ expression.

Our findings appear to confirm the hypothesis that the amyloid cascade of APP is activated in the hippocampus of patients with ALS and ALS–FTD. However, thioflavin-S staining in some of the patients revealed few intracellular or extracellular Aβ aggregates. This is consistent with *in vivo* findings from a previous study using PET with ^18^F-florbetaben, which reported low tracer uptake in the hippocampus of patients with ALS (64). This may be explained by the fact that this tracer binds to Aβ deposits and fibers and does not detect increases in peptide expression (65), as shown in our pathology study. However, some studies on ALS have shown amyloid tracer uptake (66–68). For instance, one study found tracer uptake in elderly patients (69). We hypothesize that a cohort of patients with longer survival times may present longer cascade activation periods, which may in turn lead to a greater presence of aggregates that PET imaging or thioflavin staining would detect.

Molecular changes found in the hippocampus are not linked to a specific clinical profile and progression pattern. Increased expression of AICD was only found in bulbar ALS; however, the significance of this finding is difficult to interpret. Greater expression of total or cytoplasmic TDP-43, which occurs in bulbar onset ALS and ALS associated with FTD, is correlated with poorer prognosis, meaning shorter survival time from diagnosis or shorter time elapsed to indication of mechanical ventilation. This idea is in line with studies suggesting that the measurement of TDP-43 may be a prognostic biomarker of the disease (70), although several other studies show elevations in CSF cannot be considered a sensitive diagnostic marker at that moment (71, 72).

Our study has a number of limitations. First, the included patients are representative of the most severe forms of the disease (high percentage of bulbar onset ALS and short survival time), since all of them were bodies that had been donated. We therefore cannot rule out the premise that the degree to which the amyloid cascade is activated depends on the intensity of neurodegeneration. This hypothesis is also supported by the fact that changes seemed to be more intense in patients with ALS–FTD than in those with ALS. Studies with greater sample sizes are necessary to confirm this idea. Furthermore, this limitation added to a sample of patients with short survival times does not allow us to evaluate the extent to which the amyloid cascade affects the hippocampus over longer time periods. The convenience of increase in the sample is particularly necessary in the subgroup of patients with FTD/ALS, where the small number of cases does not allow statistical analysis in any molecular marker, such as cytoplasmatic TDP-43. Second, autopsies were performed 2–6 h after death since these patients rarely die in hospital and must therefore be transported from their homes or palliative care centers. This time window may have had some impact compared with those from animal research where the times were shorter. In addition, none of the patients without FTD had cognitive alterations according to the data from medical records and clinical histories, which was confirmed by telephone interviews with the patients’ relatives. Therefore, we cannot establish a link between cognitive alterations and molecular changes in the amyloid cascade of APP in the hippocampus. This should be studied in a patient cohort with longer survival times.

In conclusion, our post-mortem analyses showed that the amyloid cascade of APP is activated in the hippocampus of patients with ALS and ALS–FTD and correlates with TDP-43 expression. Immunohistochemical analyses revealed no significant intracellular or extracellular Aβ aggregates.

## Ethical Standards

The present study complies with the ethical standards of the research committee at our center and the 1964 Declaration of Helsinki and its subsequent amendments.

## Author Contributions

Study design: UG-P and JM-G; patient evaluation: RV-Q, LG, and AG-S; coordination of autopsy studies: LG; microscopy and molecular study: UG-P and MB-M; database: UG-P and RV-Q; statistical analysis: UG-P, RV-Q, and JAM-G; analysis of results: UG-P, JAM-G, LG, TM-R, and JM-G; figures and tables: UG-P and RV-Q; manuscript draft: JM-G; manuscript revision and approval: all the authors.

## Conflict of Interest Statement

The authors declare that the research was conducted in the absence of any commercial or financial relationships that could be construed as a potential conflict of interest.

## Supplementary Material

Table S1 **Antibodies and techniques used in the study**.

Table S2 **Clinical characteristics of the patient sample**. ALS, amyotrophic lateral sclerosis; M, male; F, female; ARF, acute respiratory failure; CRA, cardiorespiratory arrest; FTD, frontotemporal dementia; PPA, primary progressive aphasia.

Table S3 **General data from the pathology study of the MND patients (numbers 4 and 7 were ALS/FTD cases)**. Semi-quantitative assessments. The scale used is 0: no alterations, +: alteration present, this alteration ++: moderately, this alteration +++: markedly.

Figure S1 **Expression of TDP-43 (A) and cytoplasmic pho-TDP-43 in mean values ± SD and individual values for controls and patients [(B,E), respectively] and fluorescence images from controls (C) and patients (D)**. Although there are no differences in total TDP-43 expression **(A)**, we found statistically significant differences (*p* = 0.0061) in the expression of cytoplasmic pho-TDP-43 **(B)**. Photomicrographs **(C,D)** show the differences in labeling between controls and patients; as can be observed, cytoplasmic pho-TDP-43 expression (arrows) is greater in patients **(D)**. **(E)** Shows the individual data corresponding to patients and controls; expression is greater in patients than in controls, and that increase is even more marked in patients with ALS–FTD. Scale bar: 50 μm. The dotted line indicates the significance threshold. The graphs present the percentage of cells with immunopositive inclusions in 500 μ^2^.

Figure S2 **The directly proportional relationship between AICD and the expression of pho-TDP-43 (A), APP (B), and Aβ (C) is displayed**.

Figure S3 **The inversely proportional connection between Fe65 and the markers of the amyloid cascade is demonstrated: APP (A), AICD (B), and Aβ peptide (C); this suggests that Fe65 levels may be downregulated by mechanisms linked to AICD and the amyloid cascade**.

Figure S4 **A direct correlation between AICD peptide and pho-TAU s396 in the hippocampus of patients with ALS (*r* = 0.570) is shown**. This is probably due to the direct link between activation of the amyloid cascade and TAU phosphorylation, which is closely related to neuronal transport. This would confirm that both AICD and pho-TAU play a major role in the pathogenesis of ALS.

Figure S5 **Correlations between total TDP-43/cytoplasmic pho-TDP-43 expression and such clinical variables as time to indication of mechanical ventilation (A), time elapsed from diagnosis to death (B), and age at death (C)**. The graph shows a weak correlation between time to mechanical ventilation and expression of pho-TDP-43 (*r* = 0.02) **(A)**. We found a linear correlation between pho-TDP-43 expression and time (in months) elapsed from diagnosis to death (*r* = 0.45) **(B)**. The correlation between total TDP-43 and age at death **(C)** was *r* = 0.47.
