# Supplementary material for: Immununochemical Markers of the Amyloid Cascade in the Hippocampus in Motor Neuron Diseases
Source: Front Neurol. 2016 Nov 8;7:195. doi: 10.3389/fneur.2016.00195 (PMC5099138; doi:10.3389/fneur.2016.00195)
Supplement: Table S1 — Antibodies and techniques used in the study. [file Table_1.PDF]

*Supplementary Material 1*

***Primary antibodies***

| ANTIGEN | IMMUNOGEN | MANUFACTURER, CATALOG, SPECIES, DILUTION USED |
|---|---|---|
| **UBIQUITIN** | RECOMBINANT FULL LENGTH PROTEIN (HUMAN) | ABCAM AB7780, RABBIT POLICLONAL ANTIBODY, DIL 1:100 |
| **TARDBP** | SYNTHETIC PEPTIDE CONJUGATED TO KLH DERIVED FROM WITHIN RESIDUES 150 - 250 OF RAT TARDBP. | ABCAM AB42474, RABBIT POLICLONAL ANTIBODY, DIL 1:100 |
| **PHOSPHO TDP-43 (Ser 409/Ser410)** | OVALBUMIN-CONJUGATED LINEAR PEPTIDE CORRESPONDING TO HUMAN TDP-43 PHOSPHORYLATED AT Ser409/Ser410 | MILLIPORE MABN14, RAT MONOCLONAL ANTIBODY, DIL. 1:100 |
| **APP** | SYNTHETIC PEPTIDE CORRESPONDING TO THE C-TERMINAL OF HUMAN APP695 (AMINO ACIDS 676-695) CONJUGATED TO KLH. | SIGMA A8717, RABBIT POLICLONAL ANTIBODY, DIL 1:1000 |
| **FE65** | GST-TAGGED RECOMBINANT PROTEIN CORRESPONDING TO HUMAN FE65. | MILLIPORE ABN451, RABBIT POLICLONAL ANTIBODY, DIL 1:200 |
| **AICD** | RECOMBINANT FULL LENGTH PROTEIN (HUMAN) | BIOLEGEND 811901, RABBIT POLICLONAL ANTIBODY, DIL 1:100 |
| **β-AMYLOID** | A SYNTHETIC PEPTIDE CONSISTING OF RESIDUES 8-17 (ser-gly-tyr-glu-val-his-his-gln-lys-leu) WITH AN ADDITIONAL C-TERMINAL CYSTEINE. | DAKO M0872, MOUSE MONOCLONAL ANTIBODY, DIL 1:100 |
| **β-AMYLOID** | SYNTHETIC PEPTIDE CORRESPONDING TO HUMAN BETA AMYLOID AA 1-14 CONJUGATED TO KEYHOLE LIMPET HAEMOCYANIN (KLH). SEQUENCE: DAEFRHDSGYEVHH | ABCAM AB2539, RABBIT POLICLONAL ANTIBODY, DIL 1:100 |
| **PHOSPHO-TAU (pSer396)** | LINEAR PEPTIDE CORRESPONDING TO HUMAN TAU PHOSPHORYLATED AT pSer396 | SIGMA SAB4504557, RABBIT POLICLONAL ANTIBODY, DIL 1:100 |
| **MAP2** | PURIFIED MICROTUBULE-ASSOCIATED PROTEIN FROM RAT BRAIN. LOT 2383071 | MILLIPORE AB5622, RABBIT POLICLONAL ANTIBODY, DIL 1:1000 |
| **NeuN** | PURIFIED CELL NUCLEI FROM MOUSE BRAIN, CLONE A60, LOT 2375608 | MILLIPORE MAB337, MOUSE MONOCLONAL ANTIBODY, DIL 1:500 |

***Secundary antibodies***

| *ANTIBODY* | *HOST* | *DILUTION USED* | *MANUFACTURER, CATALOG* |
|---|---|---|---|
| PEROXIDASE ANTI RABBIT IgG | GOAT | 1:200 | VECTOR LABS, PI-1000 |
| ALEXA FLUOR 647 ANTI RABBIT IgG | GOAT | 1:500 | INVITROGEN, A21245 |
| ALEXA FLUOR 555 ANTI RABBIT IgG | GOAT | 1:500 | INVITROGEN, A21428 |
| ALEXA FLUOR 488 ANTI RABBIT IgG | GOAT | 1:500 | INVITROGEN, A11034 |
| ALEXA FLUOR 555 ANTI RAT IgG | GOAT | 1:500 | INVITROGEN, A21434 |
| ALEXA FLUOR 488 ANTI MOUSE IgG | GOAT | 1:500 | INVITROGEN, A11029 |
