# Supplementary material for: Immununochemical Markers of the Amyloid Cascade in the Hippocampus in Motor Neuron Diseases
Source: Front Neurol. 2016 Nov 8;7:195. doi: 10.3389/fneur.2016.00195 (PMC5099138; doi:10.3389/fneur.2016.00195)
Supplement: Table S2 — Clinical characteristics of the patient sample. ALS, amyotrophic lateral sclerosis; M, male; F, female; ARF, acute respiratory failure; CRA, cardiorespiratory arrest; FTD, frontotemporal dementia; PPA, primary progressive aphasia. [file Table_2.PDF]

Supplementary Material 2.

		ALS1	ALS2	ALS3	ALS/FTD1	ALS5	ALS6	ALS/FTD2	ALS8	ALS9
**SEX**		M	F	M	M	M	M	F	F	F
**AGE AT DEATH**		37	74	70	67	60	46	67	86	81
**CAUSE OF DEATH**		ARF	ARF	ARF	ARF	ARF	ARF	CRA	ARF	ARF
**COGNITIVE IMPAIRMENT**		No	No	No	FTD 4 yr before ALS	No	No	PPA 2 yr before ALS	No	No
**FAMILY HISTORY OF COGNITIVE IMPAIRMENT**		No	Unspecified dementia	No	No	No	No	No	No	No
**ALS RELATED PARAMETERS**	**AGE AT DIAGNOSIS**	33	73	70	62	59	45	65	86	81
	**SYNTOMS TO DIAGNOSIS (MONTHS)**	6	13	2	3	11	6	6	2	9
	**SYNTOMS TO DEATH (MONTHS)**	57	27	8	10	18	17	6	3	12
	**ALS ONSET**	Spinal	Bulbar	Spinal	Bulbar	Spinal	Spinal	Bulbar	Bulbar	Bulbar
	**RILUZOLE**	Yes	Yes	Yes	Yes	Yes	Yes	No	No	Yes
	**RESPIRATORY ASSISTANT**	Yes	Yes	Yes	No	Yes	Yes	No	No	Yes
	**GASTROSTOMY**	Yes	Yes	No	No	No	Yes	Yes	No	Yes
