# Supplementary material for: Immununochemical Markers of the Amyloid Cascade in the Hippocampus in Motor Neuron Diseases
Source: Front Neurol. 2016 Nov 8;7:195. doi: 10.3389/fneur.2016.00195 (PMC5099138; doi:10.3389/fneur.2016.00195)
Supplement: Table S3 — General data from the pathology study of the MND patients (numbers 4 and 7 were ALS/FTD cases). Semi-quantitative assessments. The scale used is 0: no alterations, +: alteration present, this alteration ++: moderately, this alteration +++: markedly. [file Table_3.PDF]

Supplementary Material 3

	WEIGHT (g)	PREFRONTAL ATROPHY	ATROPHY OF ANTERIOR ROOTS	AMYLOID DEPOSITION	LOSS SPINAL MOTOR NEURON	SPINAL INCLUSIONS	HIPPOCAMPUS INCLUSIONS
**CONTROL 1**	1200	0	0	0	0	0	0
**CONTROL 2**	1360	0	0	+	0	0	0
**CONTROL 3**	1265	0	0	0	0	0	0
**CONTROL 4**	1335	0	0	0	0	0	0
**CASE 1**	1320	0	+	0	+++	0	0
**CASE 2**	1280	+	0	+	++	0	+
**CASE 3**	1520	+	0	0	++	0	0
**CASE 4**	1200	0	+	+++	+++	+	+
**CASE 5**	1220	0	0	0	+++	0	0
**CASE 6**	1000	0	0	0	+++	0	0
**CASE 7**	1200	0	0	0	++	+	+
**CASE 8**	1120	0	0	0	++	+	+
**CASE 9**	1120	0	0	0	++	+	+
