# Supplementary figures and images for: Immununochemical Markers of the Amyloid Cascade in the Hippocampus in Motor Neuron Diseases
Source: Front Neurol. 2016 Nov 8;7:195. doi: 10.3389/fneur.2016.00195 (PMC5099138; doi:10.3389/fneur.2016.00195)

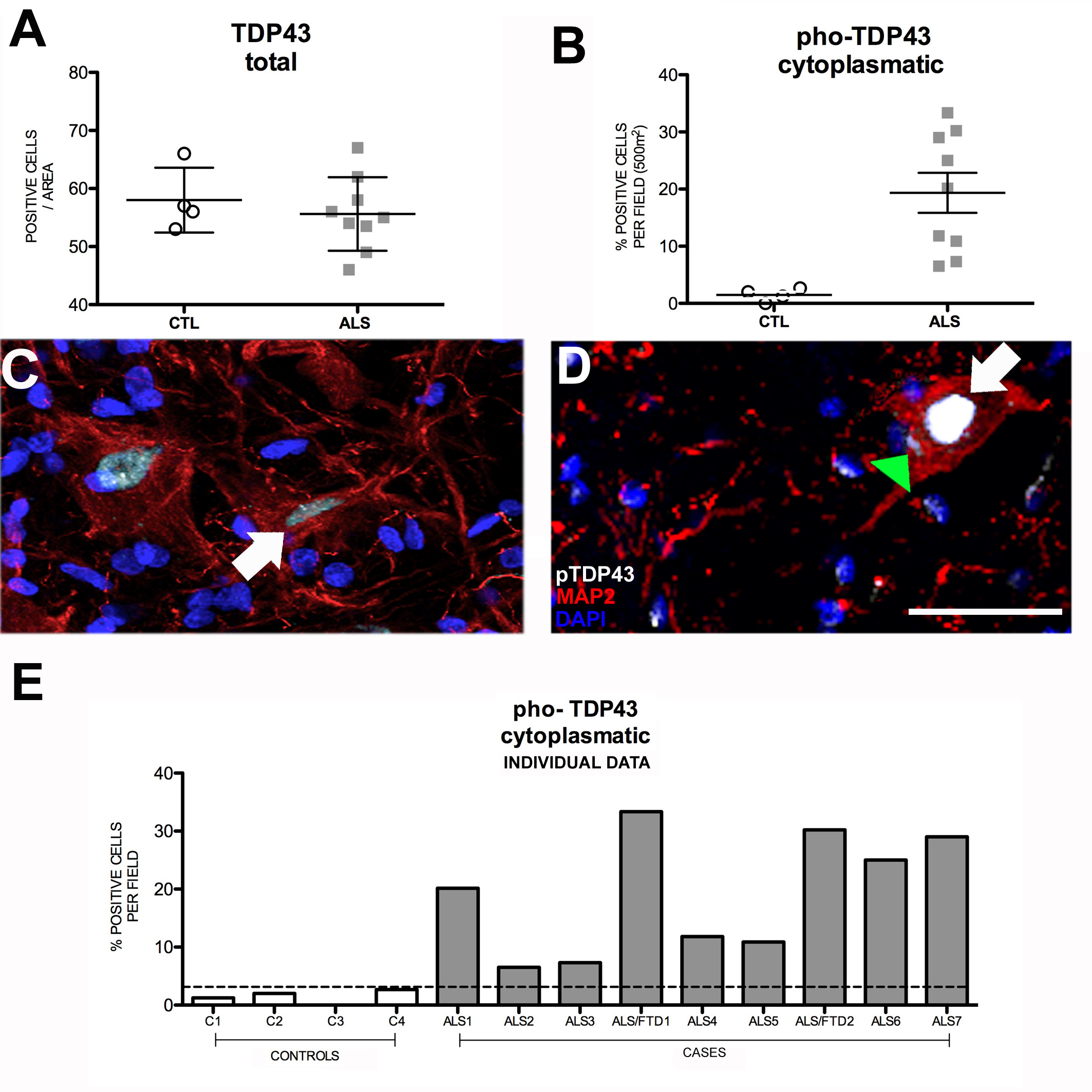

Supplement: Figure S1 — Expression of TDP-43 (A) and cytoplasmic pho-TDP-43 in mean values ± SD and individual values for controls and patients [(B,E), respectively] and fluorescence images from controls (C) and patients (D). Although there are no differences in total TDP-43 expression (A), we found statistically significant differences (p = 0.0061) in the expression of cytoplasmic pho-TDP-43 (B). Photomicrographs (C,D) show the differences in labeling between controls and patients; as can be observed, cytoplasmic pho-TDP-43 expression (arrows) is greater in patients (D). (E) Shows the individual data corresponding to patients and controls; expression is greater in patients than in controls, and that increase is even more marked in patients with ALS–FTD. Scale bar: 50 μm. The dotted line indicates the significance threshold. The graphs present the percentage of cells with immunopositive inclusions in 500 μ2. [file Image_1.jpeg]

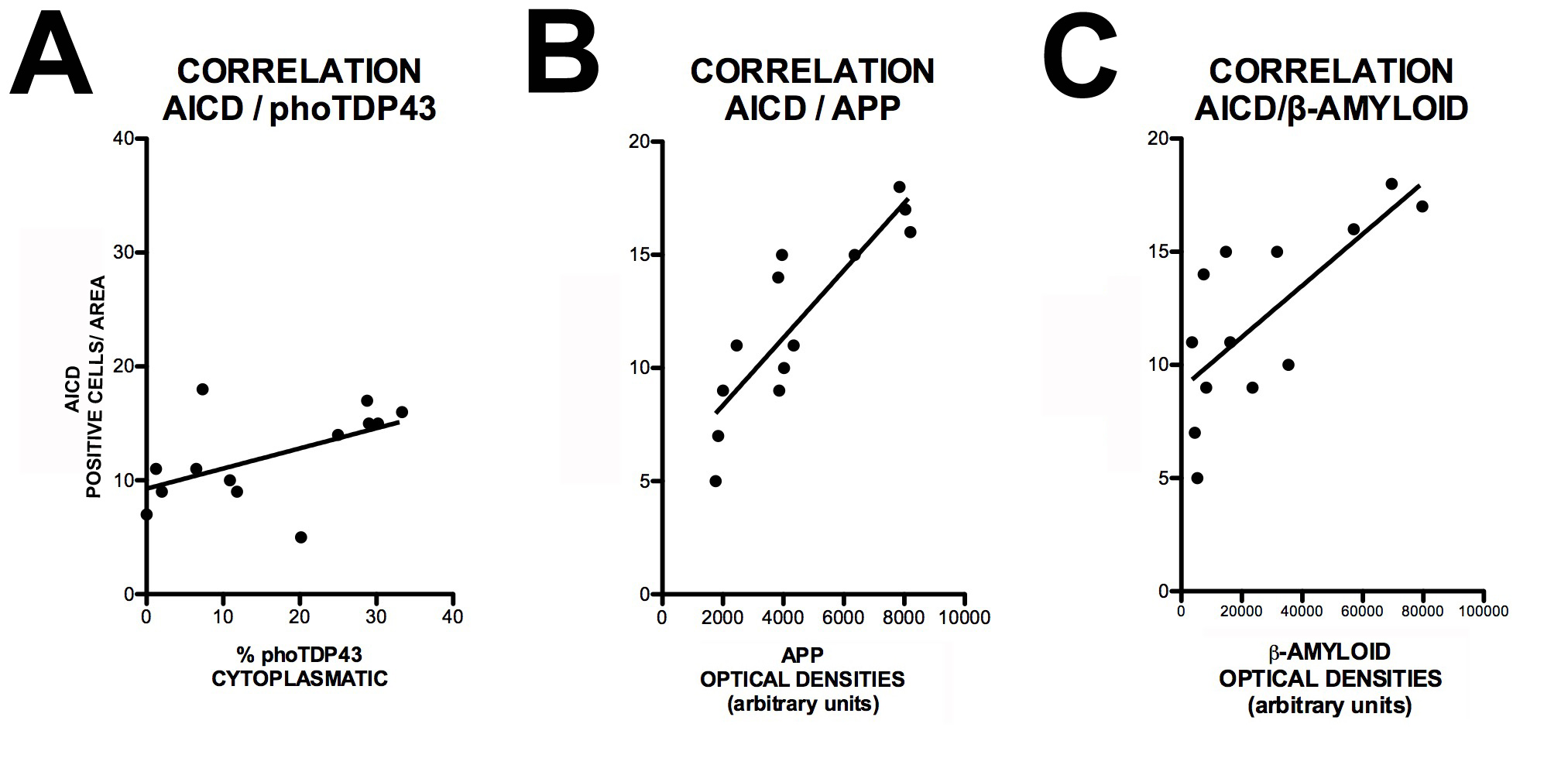

Supplement: Figure S2 — The directly proportional relationship between AICD and the expression of pho-TDP-43 (A), APP (B), and Aβ (C) is displayed. [file Image_2.jpeg]

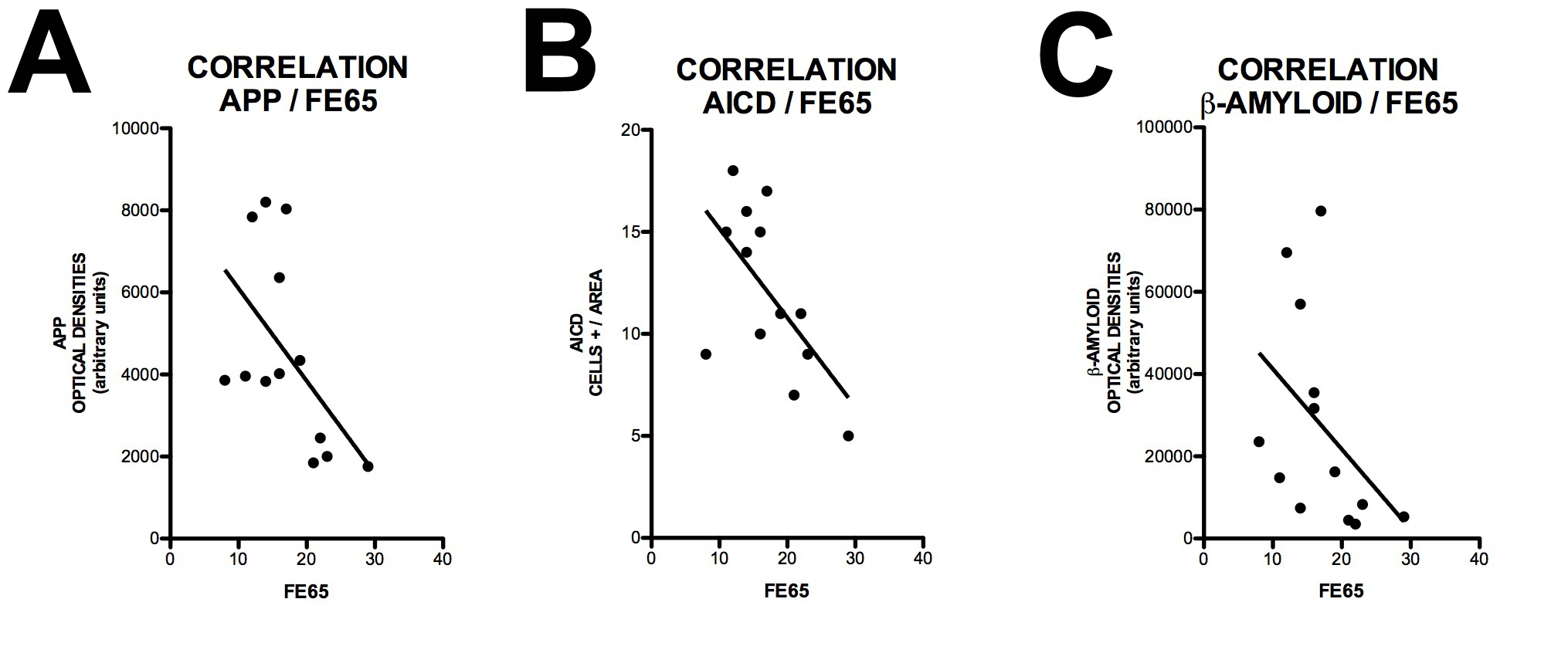

Supplement: Figure S3 — The inversely proportional connection between Fe65 and the markers of the amyloid cascade is demonstrated: APP (A), AICD (B), and Aβ peptide (C); this suggests that Fe65 levels may be downregulated by mechanisms linked to AICD and the amyloid cascade. [file Image_3.jpeg]

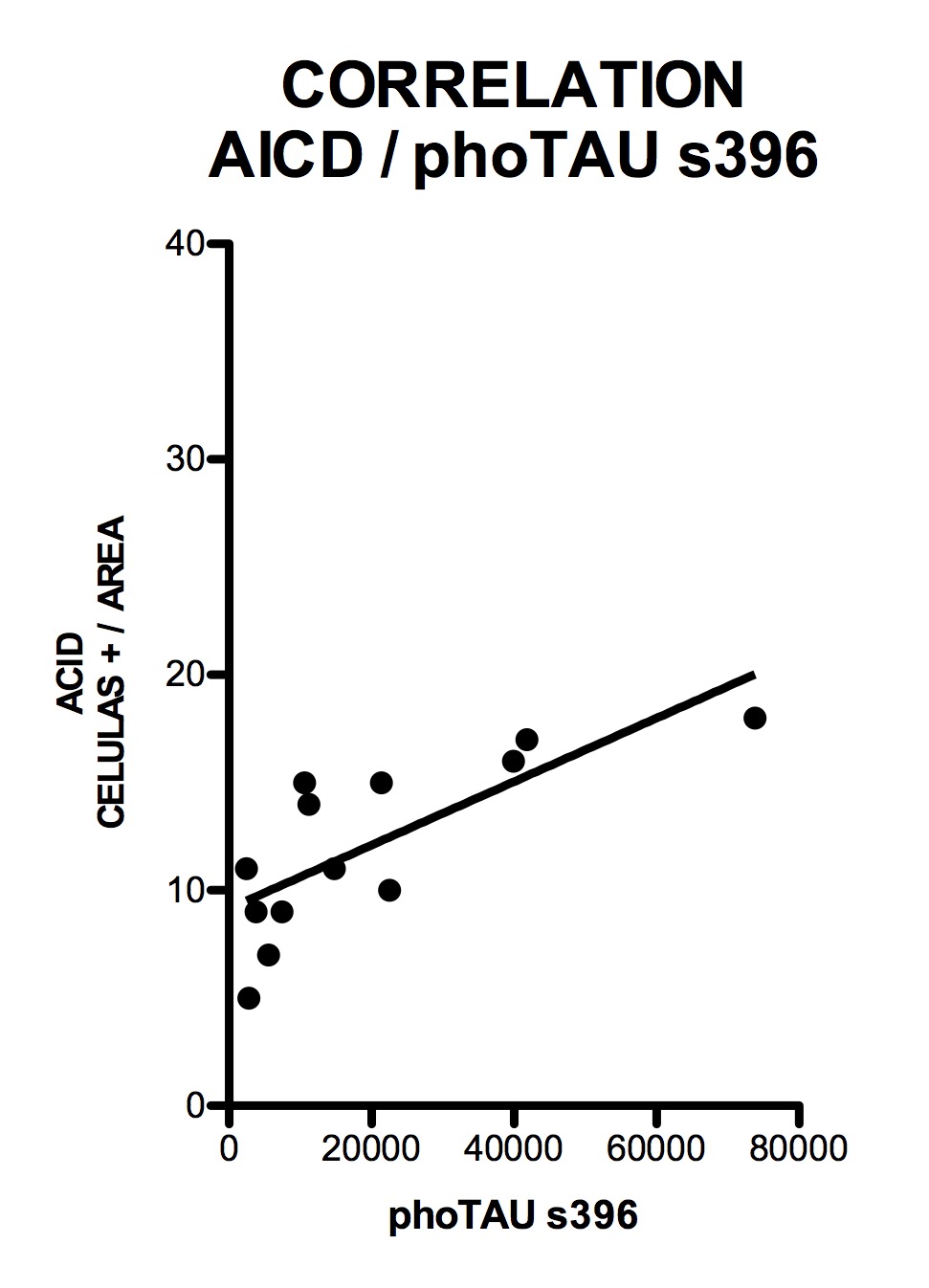

Supplement: Figure S4 — A direct correlation between AICD peptide and pho-TAU s396 in the hippocampus of patients with ALS (r = 0.570) is shown. This is probably due to the direct link between activation of the amyloid cascade and TAU phosphorylation, which is closely related to neuronal transport. This would confirm that both AICD and pho-TAU play a major role in the pathogenesis of ALS. [file Image_4.jpeg]

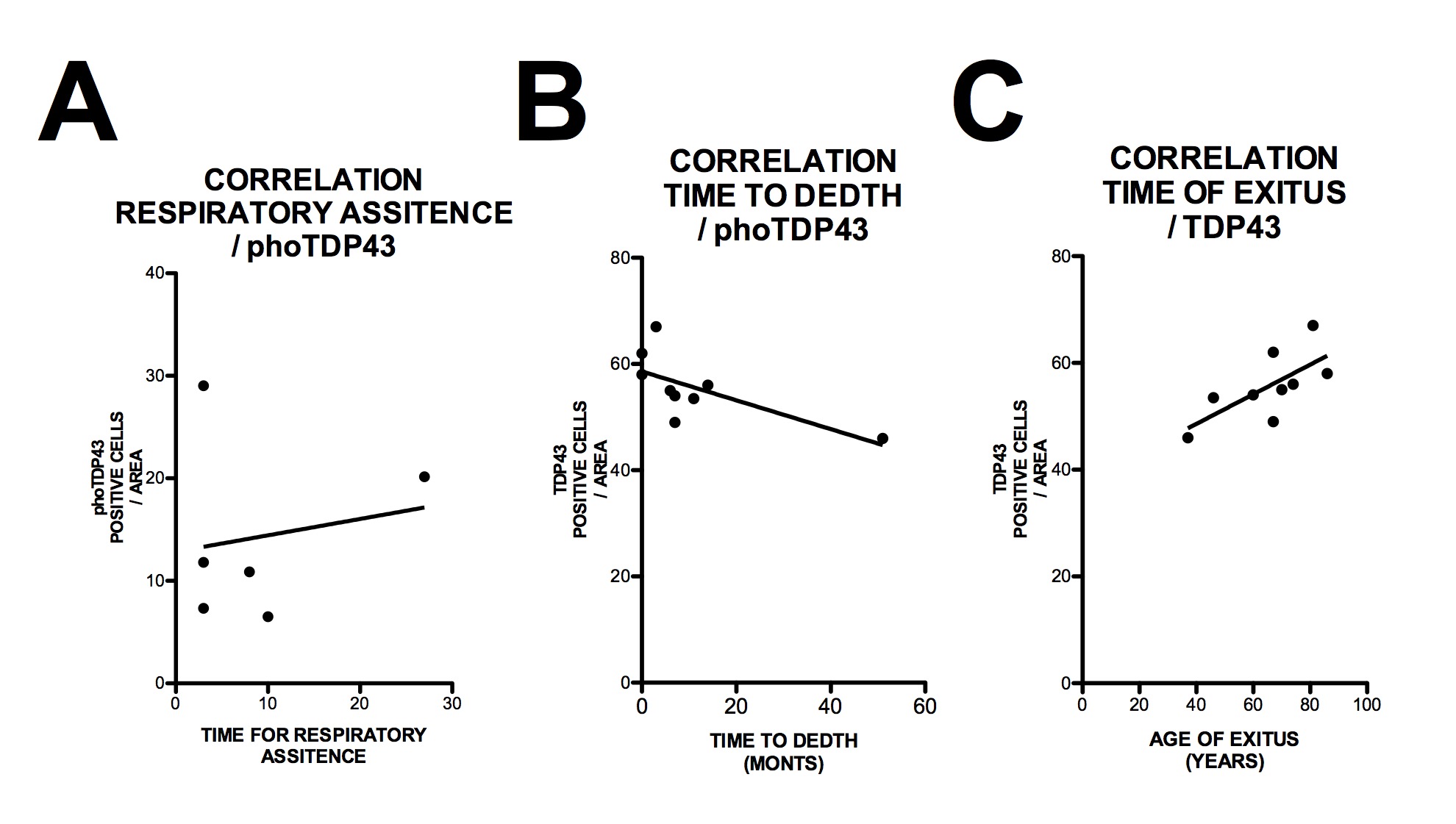

Supplement: Figure S5 — Correlations between total TDP-43/cytoplasmic pho-TDP-43 expression and such clinical variables as time to indication of mechanical ventilation (A), time elapsed from diagnosis to death (B), and age at death (C). The graph shows a weak correlation between time to mechanical ventilation and expression of pho-TDP-43 (r = 0.02) (A). We found a linear correlation between pho-TDP-43 expression and time (in months) elapsed from diagnosis to death (r = 0.45) (B). The correlation between total TDP-43 and age at death (C) was r = 0.47. [file Image_5.jpeg]
